# Supplementary material for: Parallel comparison of T cell and B cell subpopulations of adenoid hypertrophy and tonsil hypertrophy of children
Source: Nat Commun. 2025 Apr 14;16:3516. doi: 10.1038/s41467-025-58094-w (PMC11997228; doi:10.1038/s41467-025-58094-w)
Supplement: Supplementary file 1 — Supplementary Information [file 41467_2025_58094_MOESM1_ESM.pdf]

Supplementary Information for

Parallel comparison of T cell and B cell subpopulations of adenoid hypertrophy and tonsil hypertrophy of children

Yu *et al.*

This file includes:

Supplementary Figures

Supplementary Figure 1. Age and index of the peripheral blood cells of children with AH, related to Figure 1.

Supplementary Figure 2. Pathological features of AH and TH samples and overview of mNGS results, related to Figure 2.

Supplementary Figure 3. Overview of scRNA-seq data analysis for AH and TH, related to Figure 2.

Supplementary Figure 4. Fractions of major cell types in AH and TH, related to Figure 2.

Supplementary Figure 5. B cells in AH and TH, related to Figure 3.

Supplementary Figure 6. Fractions of B subtypes in AH and TH, related to Figure 3.

Supplementary Figure 7. T/NK cells in AH and TH, related to Figure 4.

Supplementary Figure 8. Fractions of T/NK cell subtypes in AH and TH, related to Figure 4.

Supplementary Figure 9. Fractions of T/NK cell subtypes in AH and TH, related to Figure 5.

Supplementary Figure 10. Cell–cell interactions between the T/NK cell subtypes and B cell subtypes. Related to Figure 7.

Supplementary Figure 11. Cell–cell interactions between the epithelial cells and T/NK cell subtypes, B cell subtypes. Related to Figure 7.

Supplementary Figure 12. Flow cytometry gating strategy for analyzing the number of naïve

CD4$^+$ T cells and the expression levels of *TP53* and *TLR4*.

Supplementary Figure 13. Flow cytometry gating strategy for analyzing the number of regulatory CD4$^+$ T cells and the expression levels of *CCL5*, *TNF*, *FOXP3* and *FOXP1*.

Supplementary Figure 14. Flow cytometry gating strategy for analyzing the number of cytotoxic CD8$^+$ T cells.

Supplementary Figure 15. Flow cytometry gating strategy for sorting B cells and T cells.

Supplementary Tables

Supplementary Table 1: Clinical variables for samples and patients used for single-cell RNA sequencing.

Supplementary Table 2: Clinical variables for samples and patients used for flow cytometry of naïve CD4$^+$ T cells and regulatory CD4$^+$ T cells.

Supplementary Table 3: Clinical variables for samples and patients used for cultivation and treatment of T cells and B cells.

Supplementary Table 4: Clinical variables for samples and patients used for flow cytometry and ELISA of cytotoxic CD8$^+$ T cells.

Supplementary Figures 1

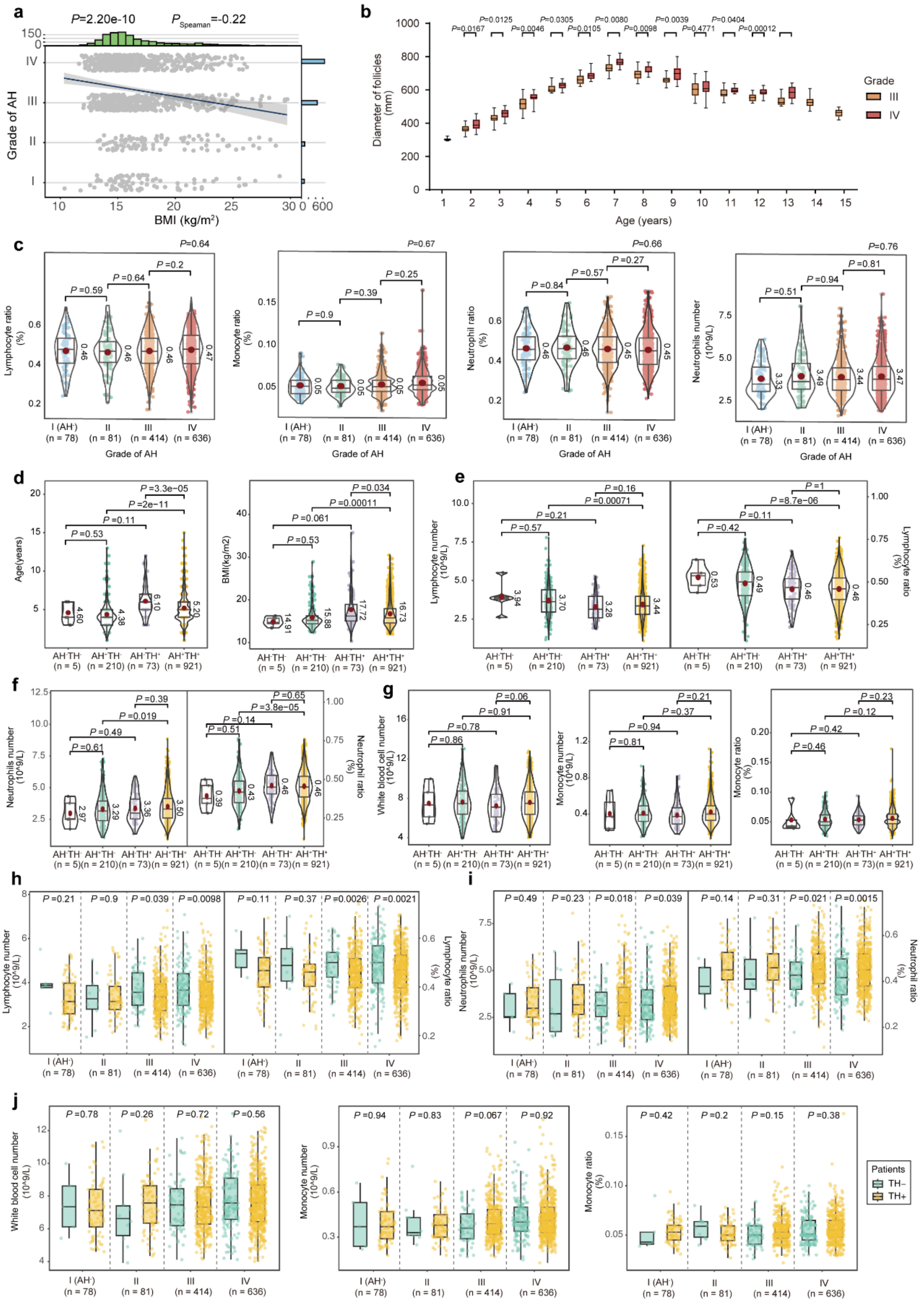

Supplementary Fig. 1 Age and index of the peripheral blood cells of children with AH, related to Figure 1.

a Scatterplot showing the correlation between the BMI (kg/m^2) in children with AH and the grade

of AH. Spearman's rank correlation coefficient (Spearman's r) was used to evaluate correlation. **b** Quantitation of follicular size. Y-axis is the number of follicles. *P* values were determined by Welch's *t*-test. **c** Violin and box plots showing lymphocytes ratio, monocytes ratio, neutrophils ratio, and neutrophils number from children with different grade of AH. Red dot represents the median value of each group. *P* values were determined by Welch's *t*-test and Welch's *F*-test. **d** Violin and box plots showing the age (years) and the BMI (kg/m^2) in children from AH^-TH^-, AH^+TH^-, AH^-TH^+, AH^+TH^+ groups. AH grade I was defined as AH^-. *P* values were determined by Welch's *t*-test. **e,f** Violin and box plots showing the number and the ratio of lymphocytes (f) and neutrophils (g) in children from AH^-TH^-, AH^+TH^-, AH^-TH^+, AH^+TH^+ groups. AH grade I was defined as AH^-. *P* values were determined by Welch's *t*-test. **g** Violin and box plots showing the number of white blood cells, the number of monocytes and the ratio of monocytes in children from AH^-TH^-, AH^+TH^-, AH^-TH^+, AH^+TH^+ group. AH grade I was defined as AH^-. *P* values were determined by Welch's *t*-test. **h,i** Box plots showing the number and the ratio of lymphocytes (i) and neutrophils (j) from children with different grade of AH with/without TH. *P* values were determined by Welch's *t*-test. **j** Box plots showing the number of white blood cells, the number of monocytes and the ratio of monocytes from children with different grade of AH with/without TH. *P* values were determined by Welch's *t*-test.

Supplementary Figures 2

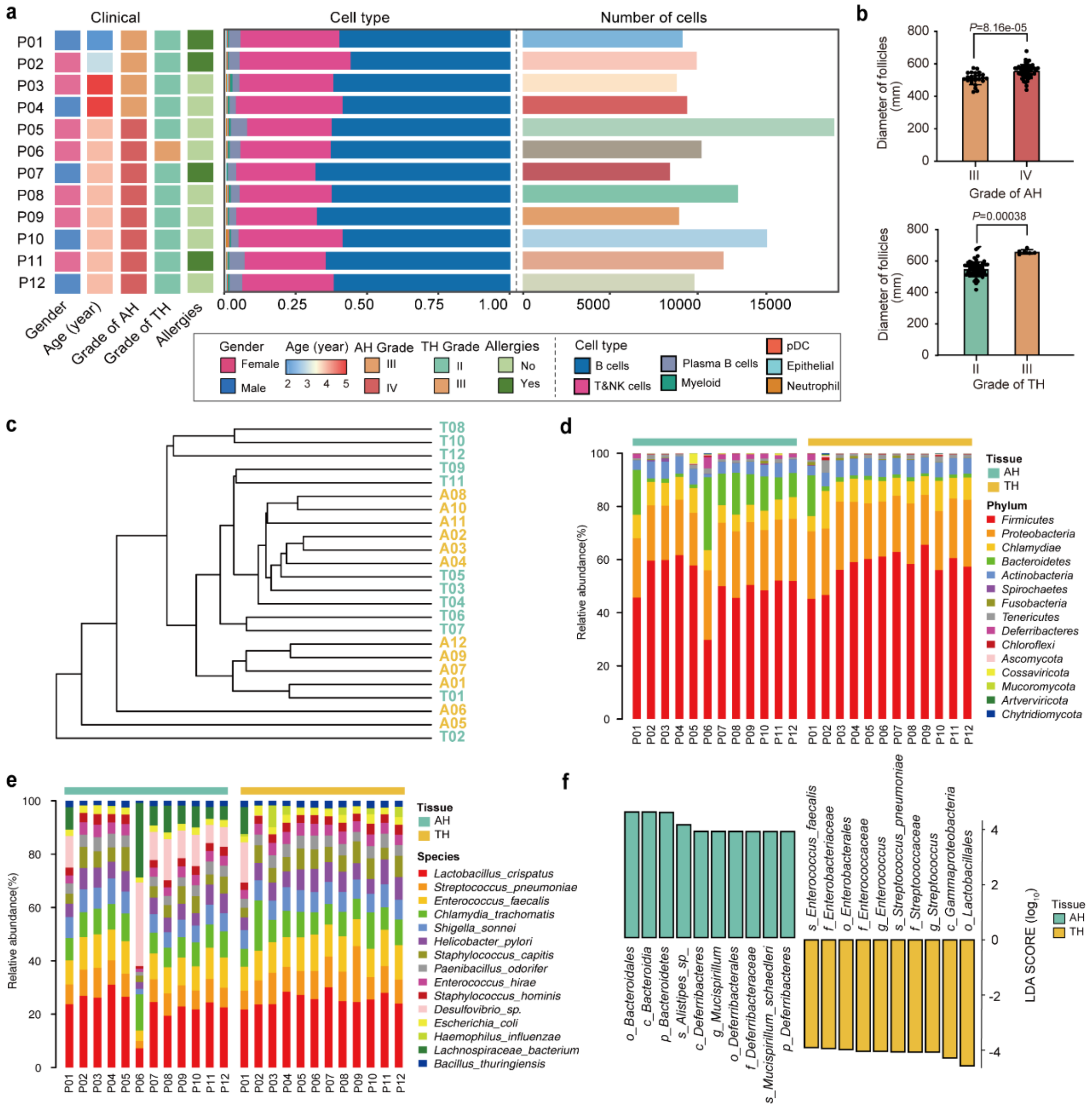

Supplementary Fig. 2 Pathological features of AH and TH samples and overview of mNGS results, related to Figure 2.

a Clinical features of the patients, the composition and number of cells in the samples. **b** Quantitation of follicular size of different grade of AH (top) and TH (bottom). Y-axis is the diameter of follicles. *P* values were determined by Welch's *t*-test. **c** Clustering tree showing the correlation of gene abundance among 24 samples. **d** Bar plot illustrating the top 15 phylum of bacteria in relative abundances of AH and TH. **e** Bar plot illustrating the top 15 species of bacteria in relative abundances of AH and TH. **f** The difference of core microbial composition between AH and TH through LEfSe difference analysis.

Supplementary Figures 3

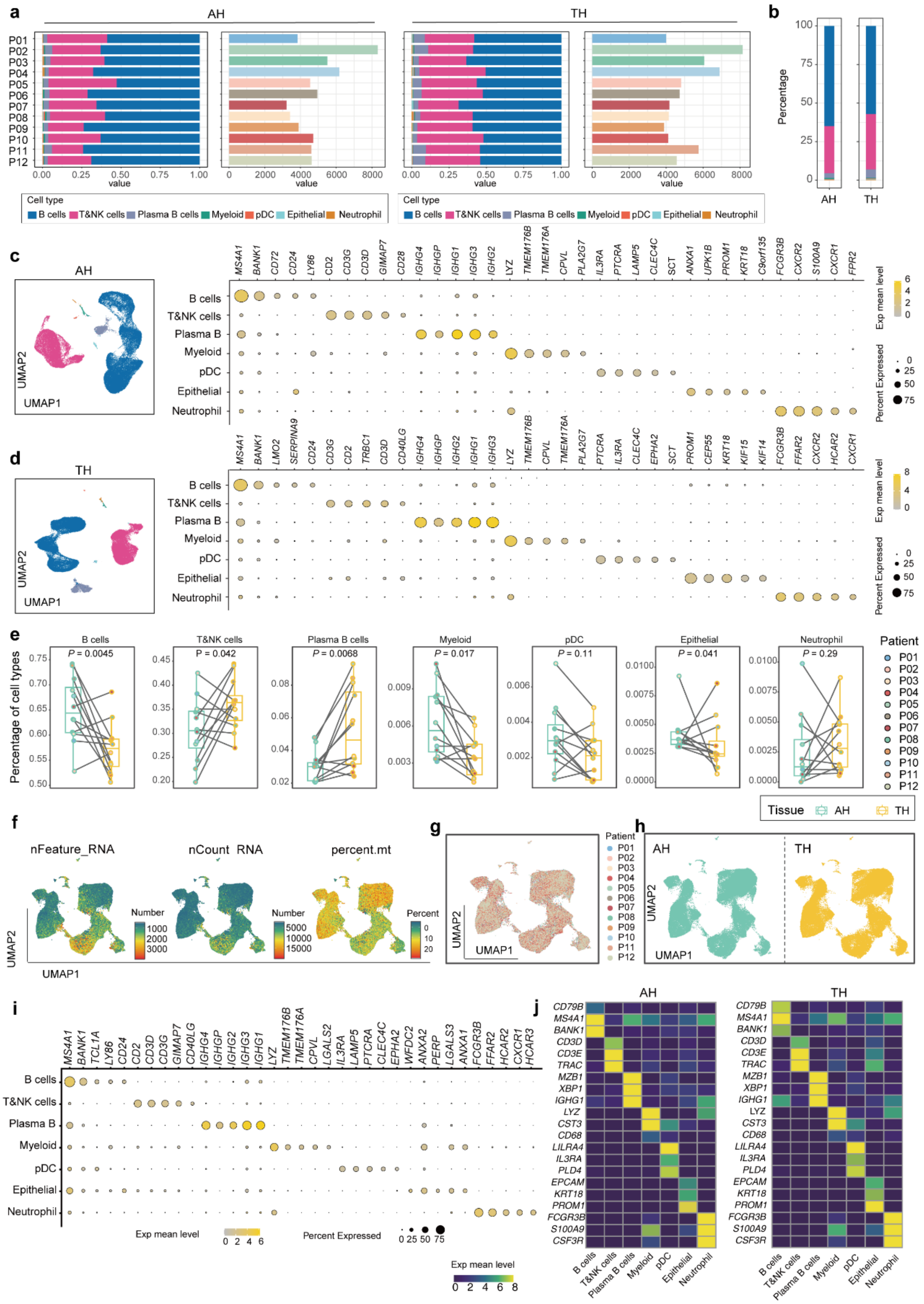

Supplementary Fig. 3 Overview of scRNA-seq data analysis for AH and TH, related to Figure 2.

a The composition and number of cells in each AH and TH sample, respectively. **b** Bar plot illustrating the fraction of major cell types in AH and TH through separate analysis of scRNA-seq

data from two tissues. B cells accounted for 64.82% and 57.18% of the total number of cells in AH and TH, T&NK cells accounted for 31.67% and 35.90%, and plasma B cells accounted for 2.98% and 5.51% in AH and TH, respectively. **c** UMAP of 63,002 cells post-QC and filtering grouped by major cell types of AH. Each dot corresponds to a single cell, colored according to cell types (left). Dot plot showing the percentage of cells and expression level in canonical cell markers of major cell types of AH. The color scheme is based on the average RNA expression distribution (right). **d** UMAP of 65,850 cells post-QC and filtering grouped by major cell types of TH. Each dot corresponds to a single cell, colored according to cell types (left). Dot plot showing the percentage of cells and expression level in canonical cell markers of major cell types of TH. The color scheme is based on the average RNA expression distribution (right). **e** Fractions of major cell types in each of the AH and TH samples through separate analysis of scRNA-seq data from two tissues (n=12). *P* values were determined by Wilcoxon's test. **f** UMAP plots showing the number of genes (nFeature) detected, number of UMIs (nCount) and percent of mitochondrial-derived transcripts (percent.mt) per single cell after quality control. **g,h** UMAP of 128,852 cells post-QC and filtering grouped by the samples (g) and the tissues (h). Each dot corresponds to a single cell, colored according to the samples (g) and the tissues (h). **i** Dot plot showing the percentage of cells and expression level in canonical cell markers of major cell types. The color scheme is based on the average RNA expression distribution. **j** Heatmap of canonical cell markers in major cell types of AH (left) and TH (right). The color scheme is based on the average RNA expression distribution.

Supplementary Figures 4

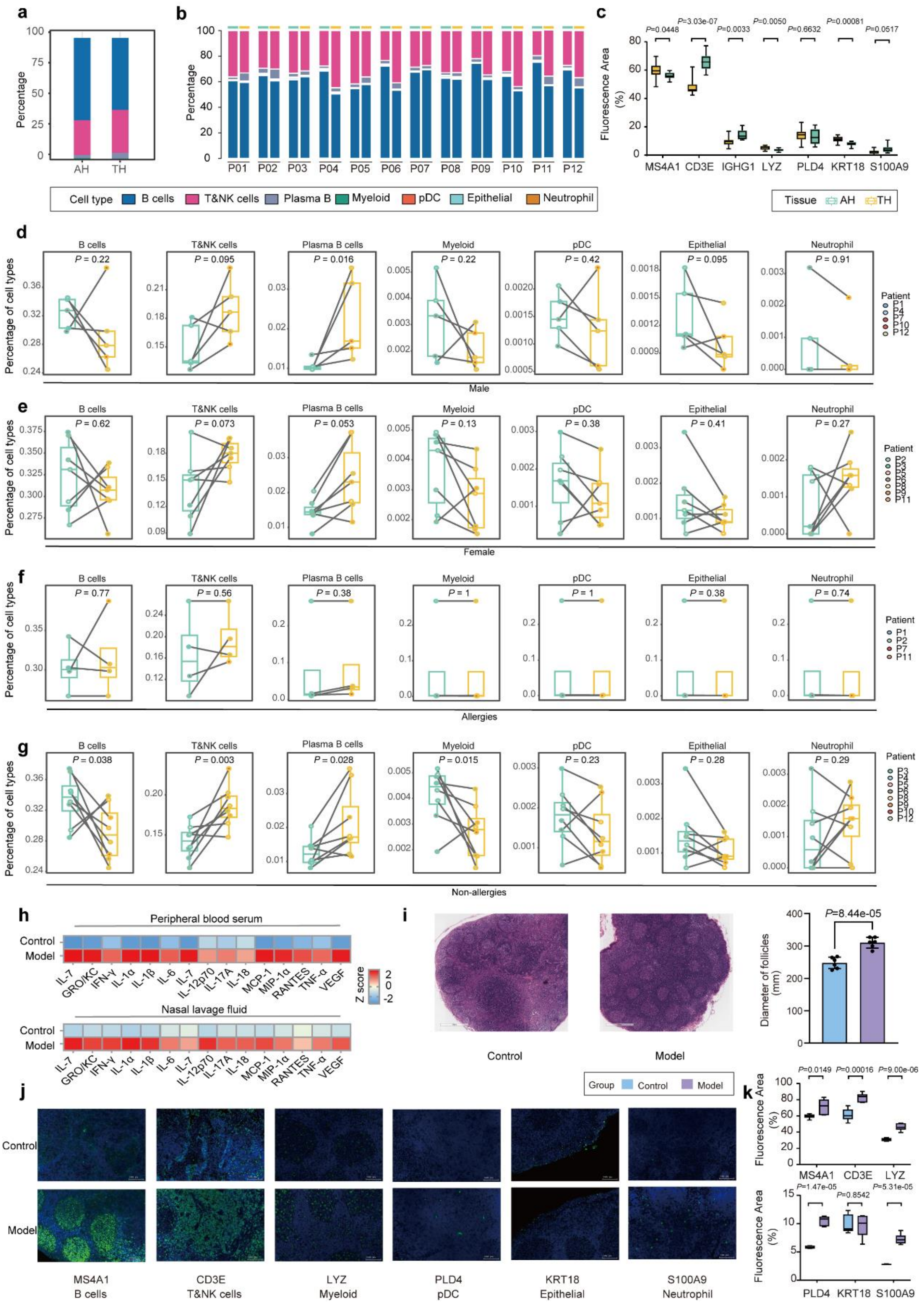

Supplementary Fig. 4 Fractions of major cell types in AH and TH, related to Figure 2.

a Bar plot illustrating the fraction of major cell types in AH and TH. **b** Bar plot indicating the proportions of cells in tissues of each patient. **c** Quantitative analysis of MS4A1, CD3E, IGHG1, LYZ, PLD4, KRT18 and S100A9 expression level by immunofluorescence images (n=12 per group).

P values were determined by Welch's *t*-test. **d-g** Fractions of major cell types in each of the AH and TH samples grouped by male (d), female (e), allergies (f) and non-allergies (g) (n=12). *P* values were determined by Wilcoxon's test. **h** Cytokine expression in peripheral blood serum and nasal lavage fluid of control and model group rats. The heatmap showing the significantly differentially expressed cytokines obtained through the Welch's *t*-test (n=6 rats/group/experiment). The color scheme is based on the scaled concentration (pg/mL). **i** Representative images of H&E-stained nasopharyngeal lymph nodes sections from control group and model group (n=6 rats/group/experiment). Scale bar: 600 μm. Quantitation of follicular size of nasopharyngeal lymph nodes in the right panel. Y-axis is the diameter of follicles. ****, $P < 0.0001$. *P* values were determined by Welch's *t*-test. **j** Representative immunofluorescence images illustrating MS4A1, CD3E, LYZ, PLD4, KRT18 and S100A9 from control group and model group (n=6 rats/group/experiment). Scale bar: 100 μm. **k** Quantitative analysis of MS4A1, CD3E, LYZ, PLD4, KRT18 and S100A9 expression level by immunofluorescence images (n=6 rats/group/experiment). *, $P < 0.05$, ***, $P < 0.001$, ****, $P < 0.0001$, ns, not significant. *P* values were determined by Welch's *t*-test.

Supplementary Figures 5

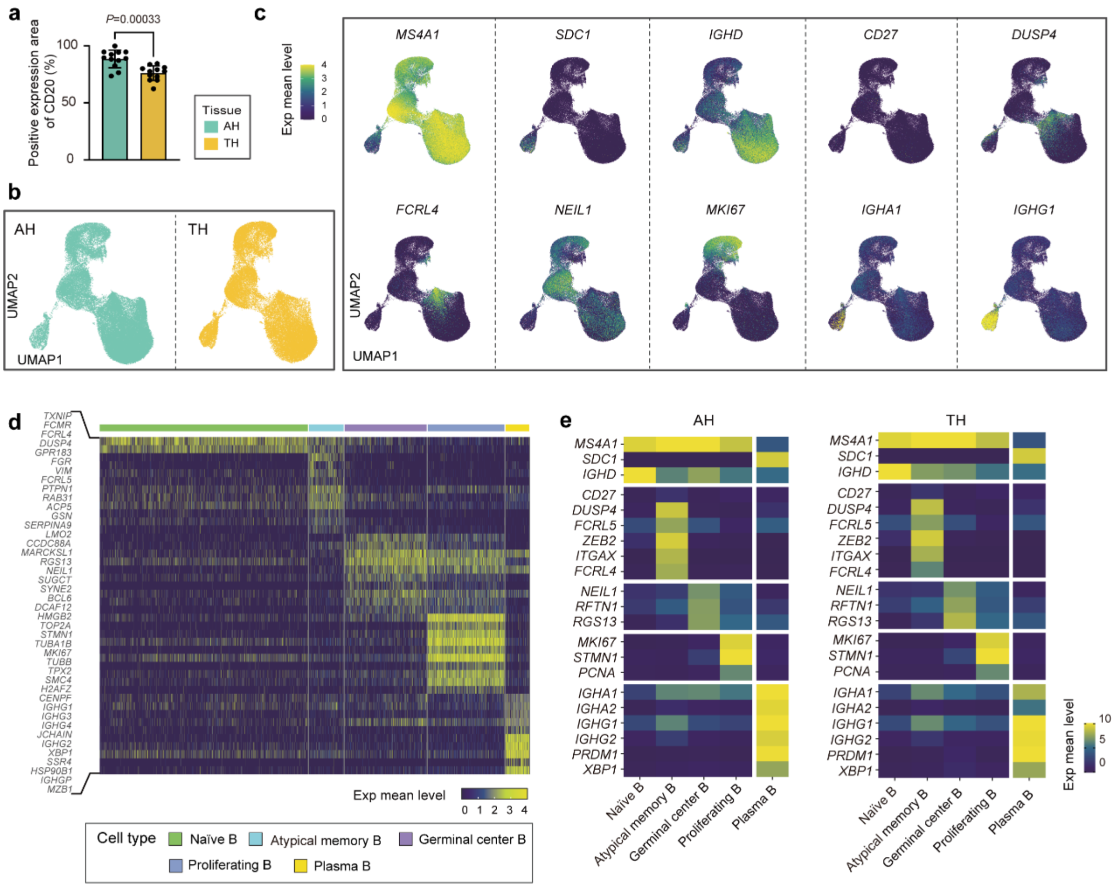

Supplementary Fig. 5 B cells in AH and TH, related to Figure 3.

a Quantitative analysis of CD20 (*MS4A1*) expression level in AH and TH by immunohistochemistry images (n=12). *P* values were determined by Welch's *t*-test. **b** UMAP plot illustrating B cells clustered and color-coded according to each tissue. **c** UMAP plots showing the expression levels of marker genes defined for the B subtypes in AH and TH. The color scheme is based on the average RNA expression distribution. **d** Heatmap of canonical cell markers in B cell subtypes. The color scheme is based on the average RNA expression distribution. **e** Heatmap of canonical cell markers in B cell subtypes of AH (left) and TH (right). The color scheme is based on the average RNA expression distribution.

Supplementary Figures 6

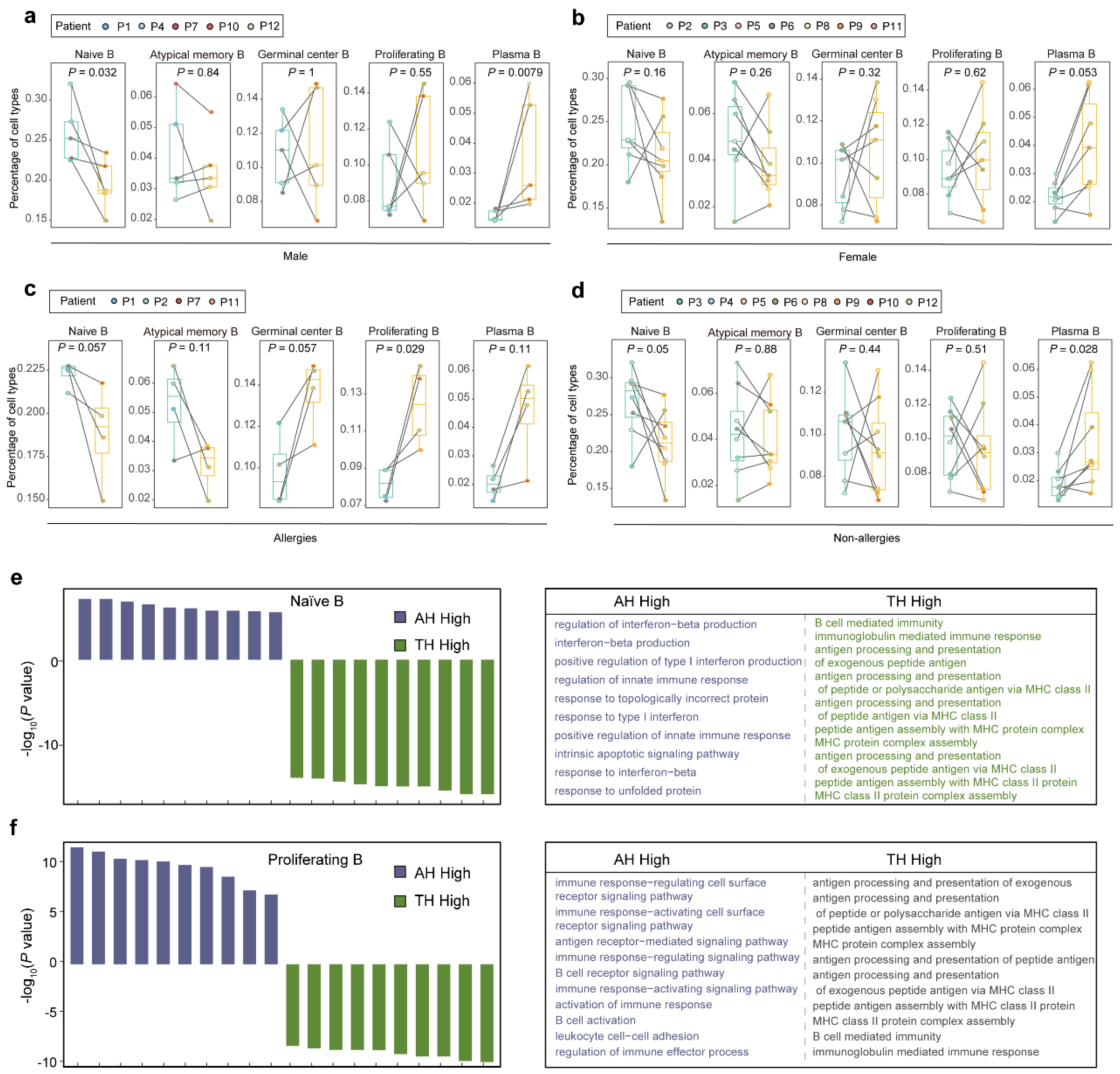

Supplementary Fig. 6 Fractions of B subtypes in AH and TH, related to Figure 3.

a-d Fractions of B subtypes in each of the AH and TH samples grouped by male (a), female (b), allergies (c) and non-allergies (d) (n=12). *P* values were determined by Wilcoxon's test. **e,f** Enriched GO terms of differentially expressed genes obtained by comparing naïve B cells (a) and proliferating B cells (b) in AH to those in TH.

Supplementary Figures 7

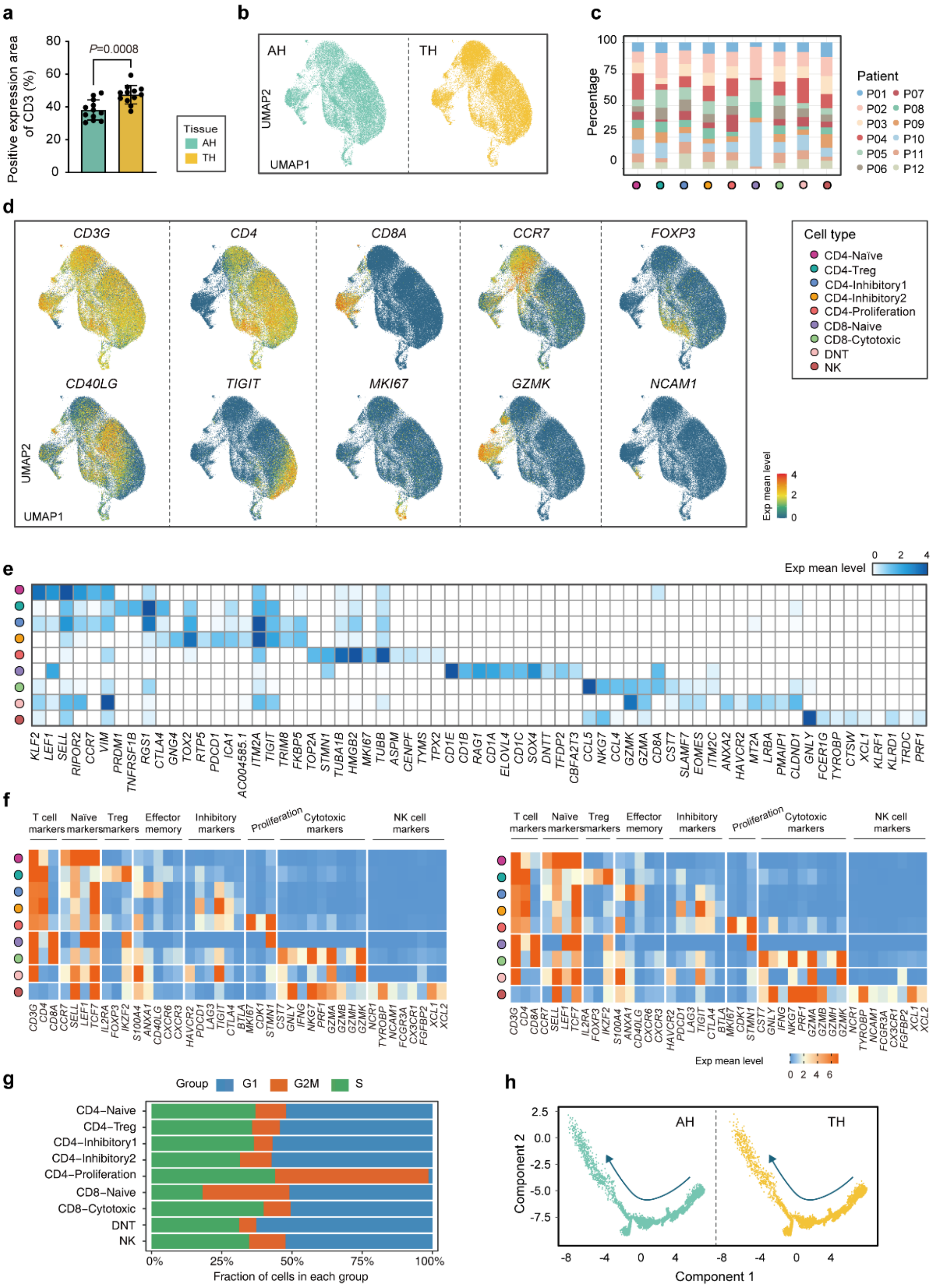

Supplementary Fig. 7 T/NK cells in AH and TH, related to Figure 4.

a Quantitative analysis of CD3 expression level in AH and TH by immunohistochemistry images (n=12). *P* values were determined by Welch's *t*-test. **b** UMAP plot illustrating T/NK cells clustered and color-coded according to each tissue. **c** Sample distribution in T/NK cell subtypes. Each bar corresponds to one cell subtype, colored according to patients. **d** UMAP plots showing the expression levels of marker genes defined for the T/NK cell subtypes in AH and TH. **e** Heatmap of

canonical cell markers in T/NK cell subtypes. **f** Heatmap of canonical cell markers in T/NK cell subtypes of AH (left) and TH (right). The color scheme is based on the average RNA expression distribution. **g** The proportion of G1, G2M and S phase cells in each T cell subtypes. **h** Differential pseudotime trajectory analysis of CD4 T cell subtypes from AH and TH samples. Tissues are labeled by colors. The arrows indicate the differentiation trajectory of the cells.

Supplementary Figures 8

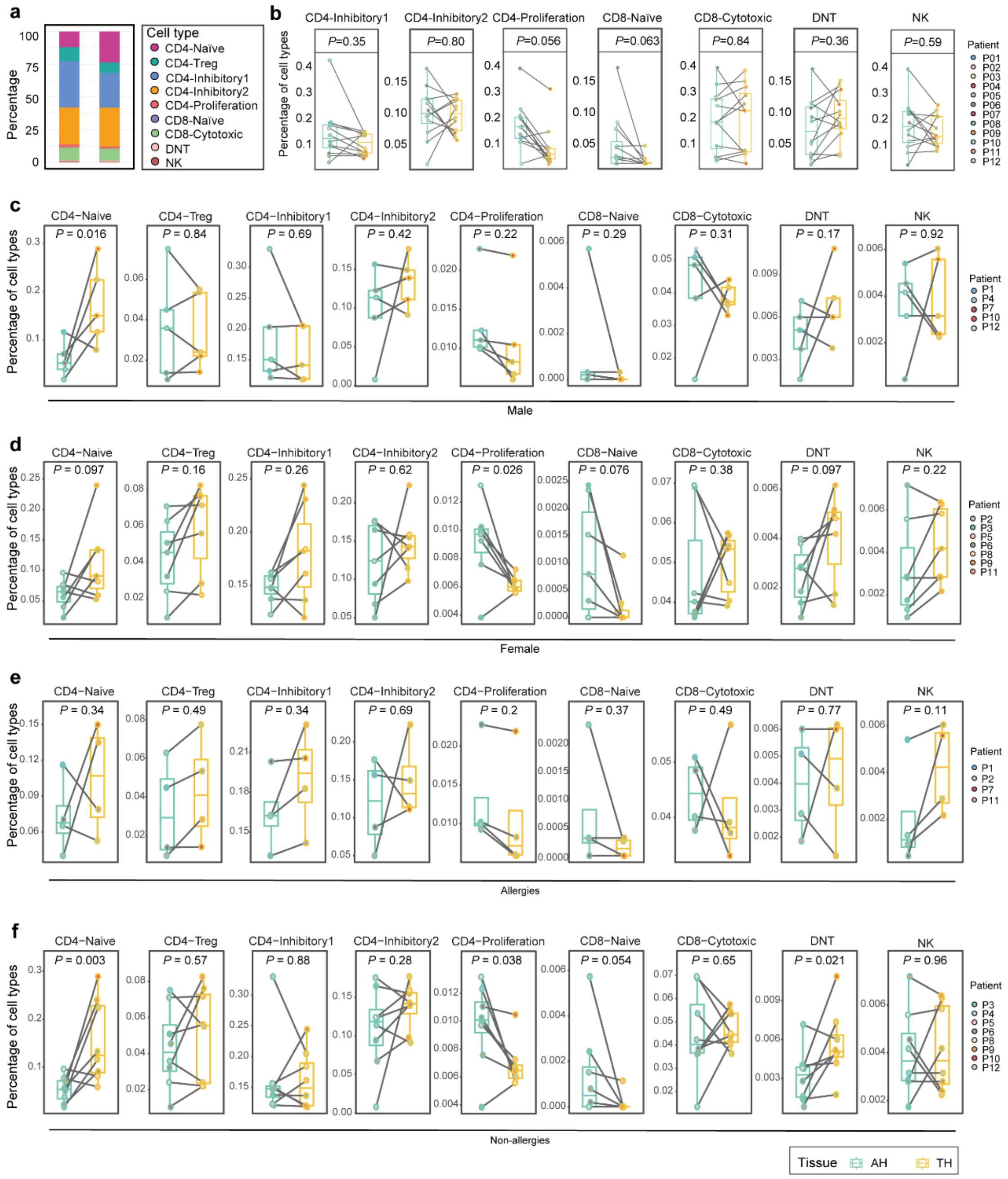

Supplementary Fig. 8 Fractions of T/NK cell subtypes in AH and TH, related to Figure 4.

a Bar plot illustrating the fraction of T/NK cell subtypes in AH and TH. **b** Fractions of exhausted effector memory CD4^{+} T cells, exhausted CD4^{+} T cells, proliferating CD4^{+} T cells, naïve CD8^{+} T cells, cytotoxic CD8^{+} T cells, double-negative T cells and NK cells in each of the AH and TH samples (n=12). *P* values were determined by Wilcoxon's test. **c-f** Fractions of T/NK cell subtypes in each of the AH and TH samples grouped by male (c), female (d), allergies (e) and non-allergies (f) (n=12). *P* values were determined by Wilcoxon's test.

Supplementary Figures 9

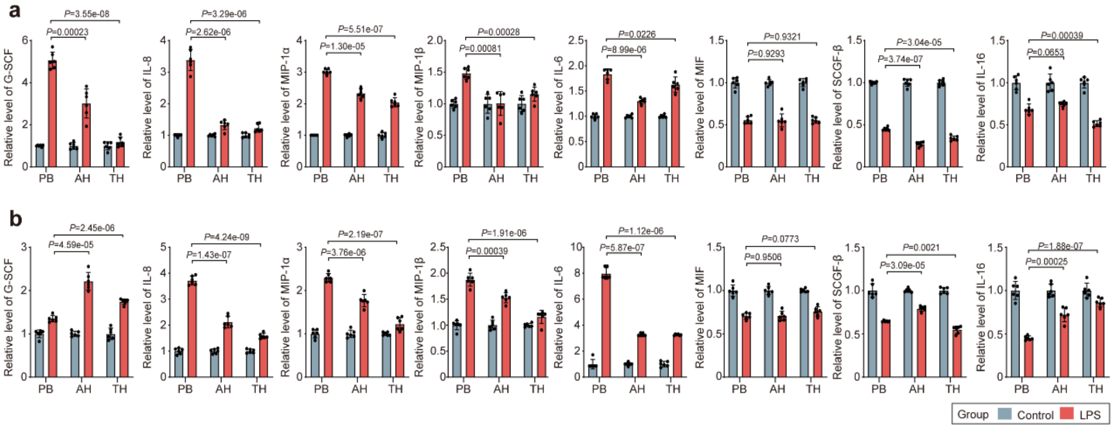

Supplementary Fig. 9 Fractions of T/NK cell subtypes in AH and TH, related to Figure 5.

a,b The relative levels of G-CSF, IL-8, MIP-1α, MIP-1β, IL-6, MIF, SCGF-β, IL-16 secreted by B (a) and T (b) cells in peripheral blood of children without inflammation, AH, and TH before and after LPS treatment. PB, peripheral blood (n=6 per group). *P* values were determined by Welch's *t*-test.

Supplementary Figures 10

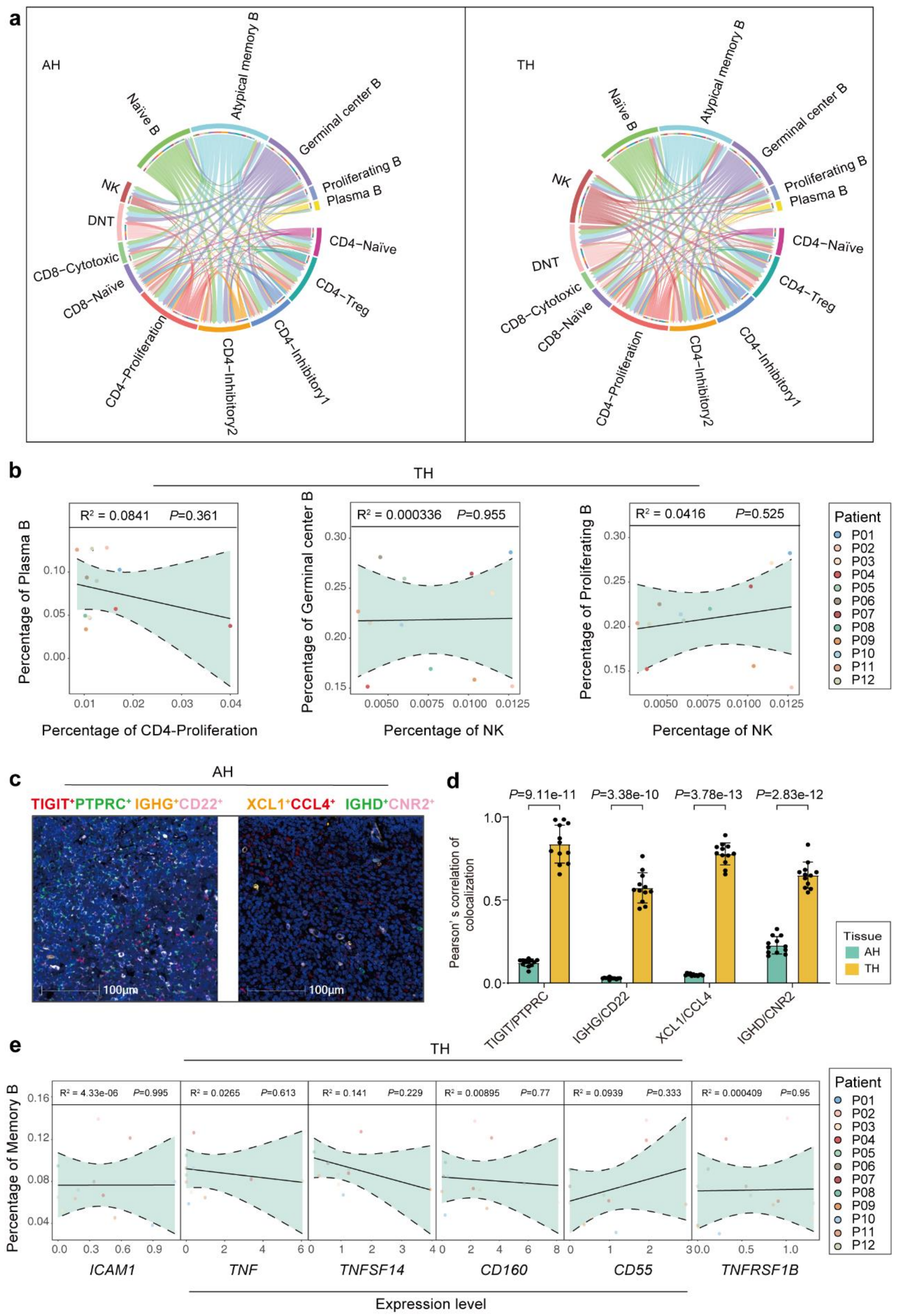

Supplementary Fig. 10 Cell–cell interactions between the T/NK cell subtypes and B cell subtypes. Related to Figure 7.

a Circos plot showing the intercellular interactions among different cell types in AH and TH. The

strings are directional and represent interactions determined on the basis of expression of a ligand by one cell types and expression of a corresponding receptor by another cell types. The thickness of each string corresponds to the amount of different interaction pairs, colored according to cell types. **b** Scatterplot showing the correlation between the relative ratio of proliferating B cells and plasma B cells (left), NK cells and germinal centre B cells (middle), NK cells and proliferating B cells (right) from TH in the scRNA-seq dataset. Pearson's correlation coefficient (Pearson's r) was used to evaluate correlation. **c** Representative immunofluorescence images illustrating the PTPRC expression in exhausted $CD4^+$ T ($TIGIT^+$) cells, the CD22 expression in plasma B cells ($IGHG1^+$) in one AH sample (P6) (left), the CCL4 expression in NK cells ($XCL1^+$) and the CNR2 expression in naïve B cells ($IGHD^+$) in one AH sample (P2) (right). Scale bar: 100 µm. **d** Pearson's correlation of fluorescence co-localization between AH and TH groups calculated by image J software (n=12). *P* values were determined by Welch's *t*-test. **e** Scatterplot showing the correlation between the expression of *ICAM1*, *TNF*, *TNFSF14*, *CD160*, *CD55*, *TNFRSF1B* in NK cells and the proportion of atypical memory B cells from TH, respectively. Pearson's correlation coefficient (Pearson's r) was used to evaluate correlation.

Supplementary Figures 11

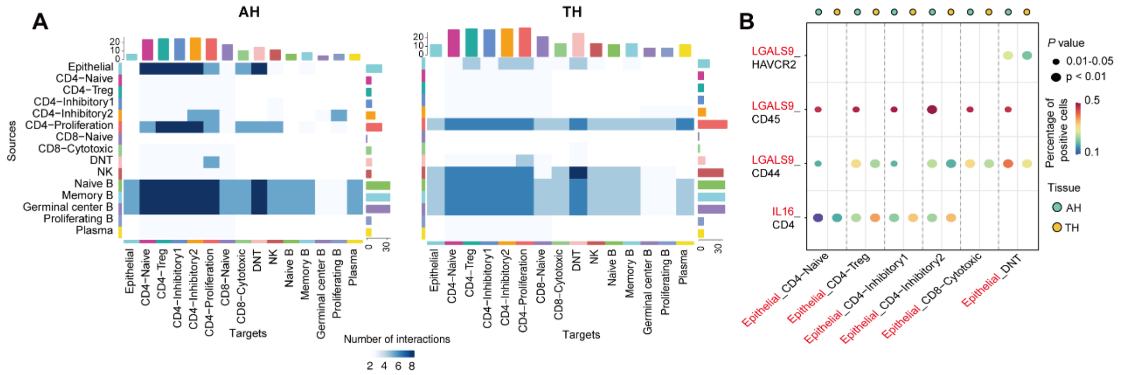

Supplementary Fig. 11 Cell–cell interactions between the epithelial cells and T/NK cell subtypes, B cell subtypes. Related to Figure 7.

a Heatmap showing the number of intercellular interactions among different cell types in AH and TH. The vertical axis represents cell types containing ligands, while the horizontal axis represents cell types containing receptors. The color scheme is based on the number of intercellular interactions.

b Dot plot showing intercellular interactions between epithelial cells and CD4-Naïve, CD4-Treg, CD4-Inhibitory1, CD4-Inhibitory2, CD8-Cytotoxic, DNT. Circle size indicates the significance of the interaction, and circle color indicates the percentage of genes expressing the ligand receptor pair. The red letters represent ligands, and the black letters represent receptors.

Supplementary Figures 12

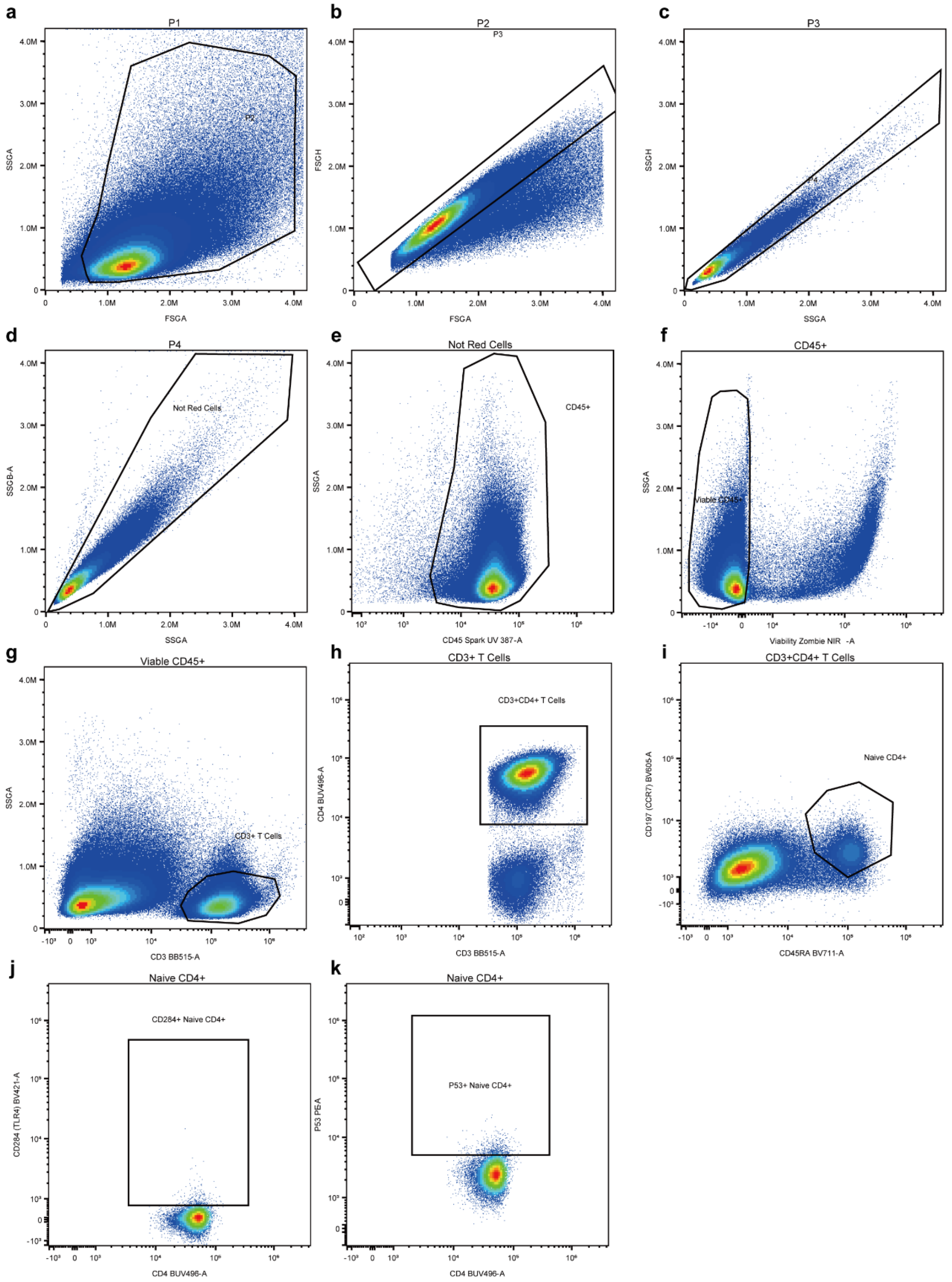

Supplementary Fig. 12 Flow cytometry gating strategy for analyzing the number of naïve CD4^{+} T cells and the expression levels of *TP53* and *TLR4.*

a Using FSC and SSC parameters to remove debris from single cells and selecting complete clusters of target cells. **b** Exclude doublets from single cells through the area and height of FSC. **c** Exclude doublets from single cells through the area and height of SSC. **d** Remove red cells from the target

cells. **e** Select all white blood cells using CD45 antibody. **f** Select all living white blood cells. **g** Select T cells using T cell specific antibody CD3. **h** Select $CD4^+$ T cells using CD4 antibody. **i** Select naïve $CD4^+$ T cells using CD45RA and CD197 antibody. **j** Select naïve $CD4^+$ T cells expressing CD284 (*TLR4*) to analyze cell percentage and CD284 (*TLR4*) expression intensity. **k** Select naïve $CD4^+$ T cells expressing P53 (*TP53*) to analyze cell percentage and P53 (*TP53*) expression intensity.

Supplementary Figures 13

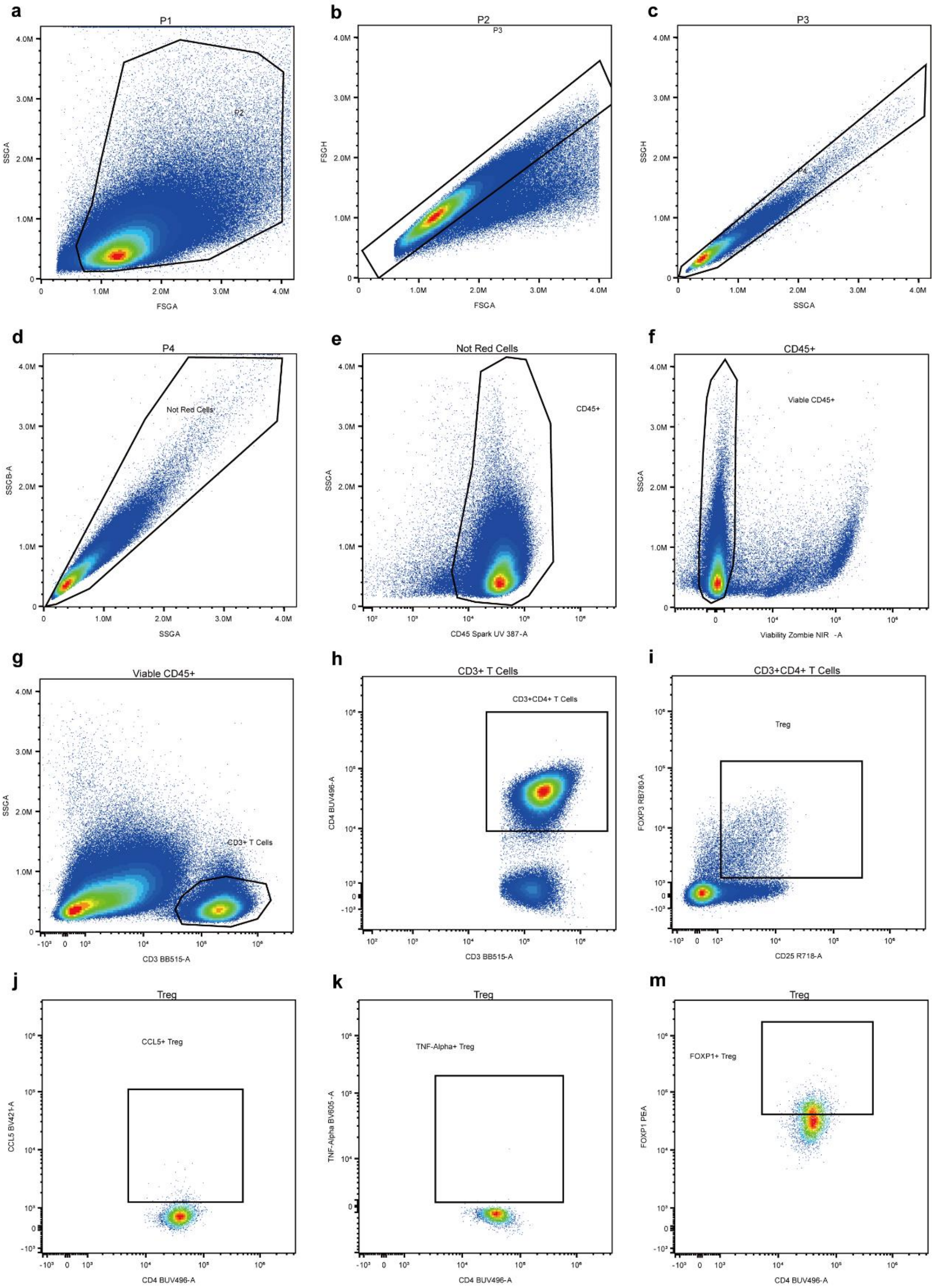

Supplementary Fig. 13 Flow cytometry gating strategy for analyzing the number of regulatory CD4^{+} T cells and the expression levels of *CCL5*, *TNF*, *FOXP3* and *FOXP1*.

a Using FSC and SSC parameters to remove debris from single cells and selecting complete clusters of target cells. **b** Exclude doublets from single cells through the area and height of FSC. **c** Exclude doublets from single cells through the area and height of SSC. **d** Remove red cells from the target

cells. **e** Select all white blood cells using CD45 antibody. **f** Select all living white blood cells. **g** Select T cells using T cell specific antibody CD3. **h** Select CD4$^+$ T cells using CD4 antibody. **i** Select regulatory CD4$^+$ T cells using CD25 and FOXP3 antibody to analyze cell percentage and FOXP3 expression intensity. **j** Select regulatory CD4$^+$ T cells expressing CCL5 to analyze cell percentage and CCL5 expression intensity. **k** Select regulatory CD4$^+$ T cells expressing TNF to analyze cell percentage and TNF expression intensity. **m** Select regulatory CD4$^+$ T cells expressing FOXP1 to analyze cell percentage and FOXP1 expression intensity.

Supplementary Figures 14

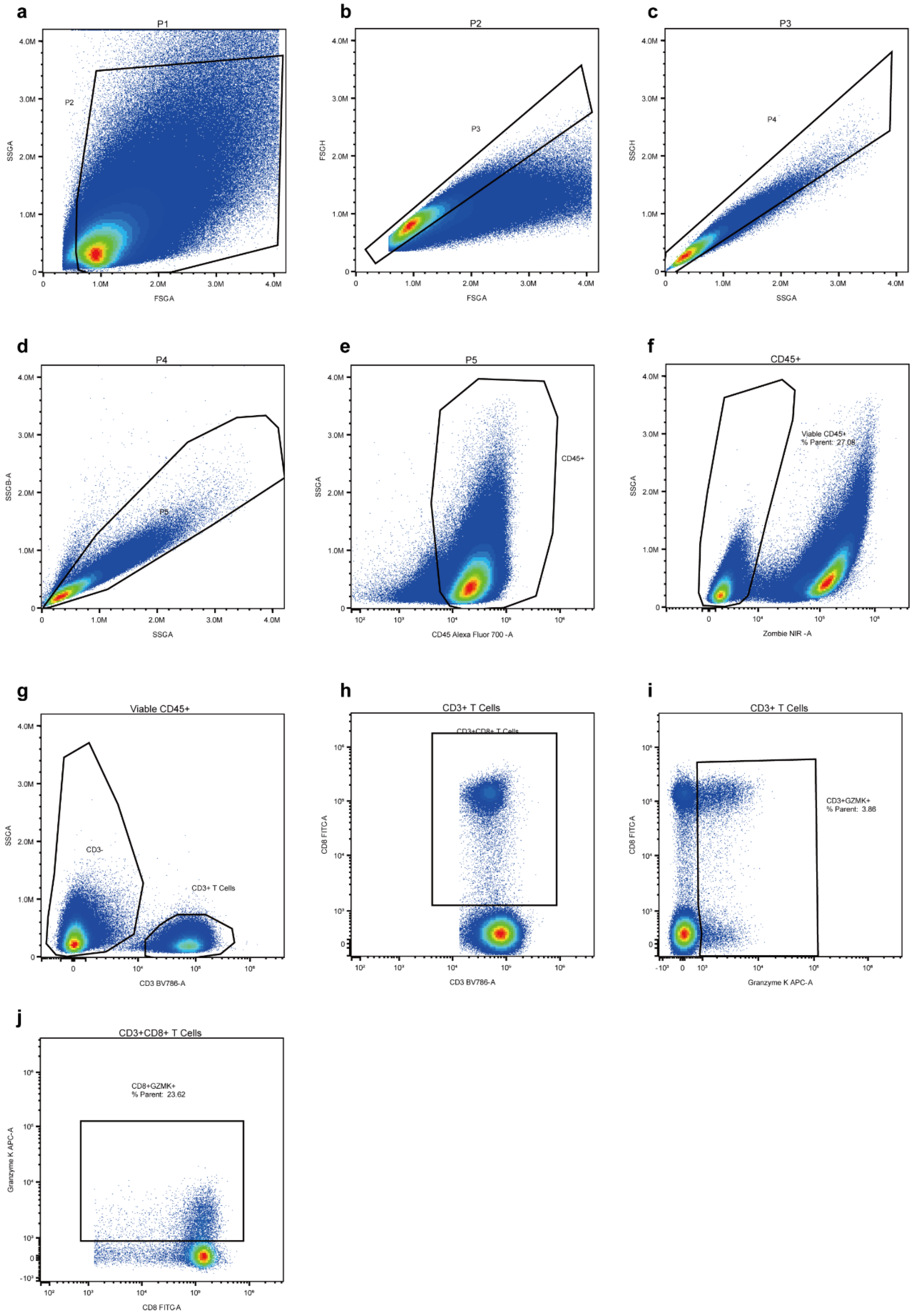

Supplementary Fig. 14 Flow cytometry gating strategy for analyzing the number of cytotoxic CD8⁺ T cells.

a Using FSC and SSC parameters to remove debris from single cells and selecting complete clusters of target cells. **b** Exclude doublets from single cells through the area and height of FSC. **c** Exclude

doublets from single cells through the area and height of SSC. **d** Remove red cells from the target cells. **e** Select all white blood cells using CD45 antibody. **f** Select all living white blood cells. **g** Select T cells using T cell specific antibody CD3. **h** Select $CD8^+$ T cells using CD8 antibody. **i** Select T cells expressing GZMK using CD3 and GZMK antibody. **j** Select cytotoxic $CD8^+$ T cells using CD8 and GZMK antibody to analyze cell percentage.

Supplementary Figures 15

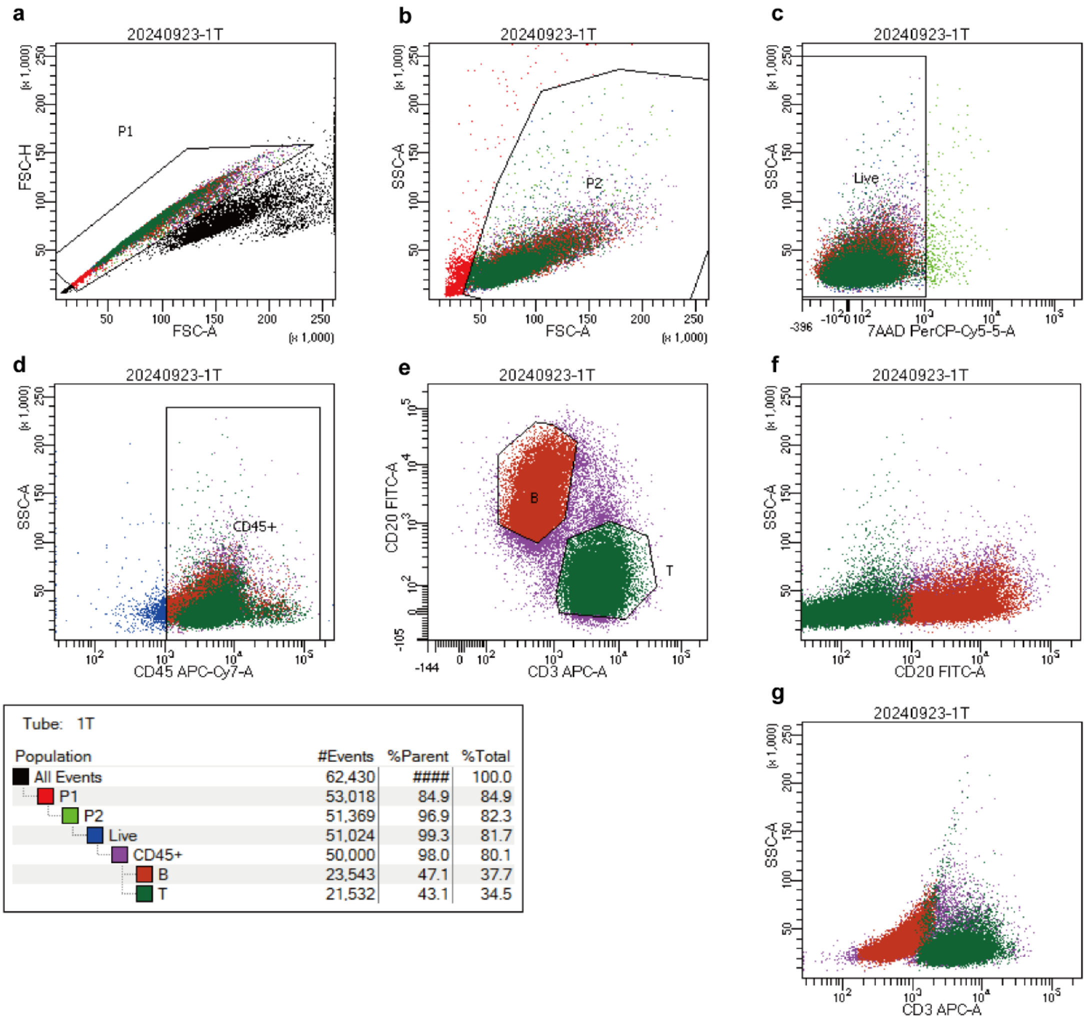

Population	#Events	%Parent	%Total
All Events	62,430	####	100.0
P1	53,018	84.9	84.9
P2	51,369	96.9	82.3
Live	51,024	99.3	81.7
CD45+	50,000	98.0	80.1
B	23,543	47.1	37.7
T	21,532	43.1	34.5

Supplementary Fig. 15 Flow cytometry gating strategy for sorting B cells and T cells.

a Exclude doublets from single cells through the area and height of FSC. **b** Using FSC and SSC parameters to remove debris from single cells and selecting complete clusters of target cells. **c** Remove dead cells from the target cells. **d** Select all white blood cells using CD45 antibody in live cells. **e,f** Select T cells and B cells using T cell specific antibody CD3 (e) and B cell specific antibody CD20 (f), respectively.

Supplementary Table 1: Clinical variables for samples and patients used for single-cell RNA sequencing.

Surgery Date	Patient ID	Gender	Age	Grade of AH	Grade of TH	Pneumococcal Vaccination	Allergies	Smoke Exposure
210419	P01	M	2	III	II	Yes	Wormwood	No
210607	P02	F	3	III	II	Yes	Wormwood	No
210628	P03	F	5	III	II	Yes	No	No
210705	P04	M	5	III	II	Yes	No	No
210419	P05	F	4	IV	II	Yes	No	No
210607	P06	F	4	IV	III	Yes	No	No
210628	P07	M	4	IV	II	Yes	Dog Hair	No
210520	P08	F	4	IV	II	Yes	No	No
210705	P09	F	4	IV	II	Yes	No	No
210520	P10	M	4	IV	II	Yes	No	No
210719	P11	F	4	IV	II	Yes	Mould	No
210719	P12	M	4	IV	II	Yes	No	No

Supplementary Table 2: Clinical variables for samples and patients used for flow cytometry of naïve $CD4^+$ T cells and regulatory $CD4^+$ T cells.

Surgery Date	Patient ID	Gender	Age	Grade of AH	Grade of TH
241014	P01	M	4	III	II
241014	P02	M	4	III	II
241014	P03	M	4	III	II
241021	P04	F	5	IV	II
241021	P05	F	5	IV	II
241021	P06	M	6	III	II
241104	P07	M	3	IV	II
241104	P08	M	3	IV	II
241107	P09	F	5	IV	II

Supplementary Table 3: Clinical variables for samples and patients used for cultivation and treatment of T cells and B cells.

Surgery Date	Patient ID	Gender	Age	Grade of AH	Grade of TH
240923	P01	M	3	III	II
240923	P02	M	3	IV	II
240923	P03	M	4	III	II
240923	P04	M	5	IV	II
240923	P05	M	6	IV	II
240923	P06	F	7	III	II

Supplementary Table 4: Clinical variables for samples and patients used for flow cytometry and ELISA of cytotoxic $CD8^+$ T cells.

Surgery Date	Patient ID	Gender	Age	Grade of AH
231219	P01	F	4	IV
231219	P02	M	4	IV
231219	P03	M	4	III
231219	P04	M	4	IV
231221	P05	M	3	IV
231221	P06	M	4	III
231221	P07	F	5	IV
240102	P08	M	4	III
240102	P09	M	4	IV
240102	P10	M	6	IV
240102	P11	F	3	III
240102	P12	F	3	III
240104	P13	M	4	IV
240104	P14	F	4	IV
240109	P15	M	4	III
240109	P16	M	4	III
240111	P17	F	4	III
240112	P18	F	6	III
