# Supplementary material for: Parallel comparison of T cell and B cell subpopulations of adenoid hypertrophy and tonsil hypertrophy of children
Source: Nat Commun. 2025 Apr 14;16:3516. doi: 10.1038/s41467-025-58094-w (PMC11997228; doi:10.1038/s41467-025-58094-w)
Supplement: Supplementary file 2 — Reporting Summary [file 41467_2025_58094_MOESM2_ESM.pdf]

nature portfolio

Corresponding author(s): Jing Yuan

Last updated by author(s): Feb 7, 2025

Reporting Summary

Nature Portfolio wishes to improve the reproducibility of the work that we publish. This form provides structure for consistency and transparency in reporting. For further information on Nature Portfolio policies, see our Editorial Policies and the Editorial Policy Checklist.

Statistics

For all statistical analyses, confirm that the following items are present in the figure legend, table legend, main text, or Methods section.

n/a	Confirmed	
☐	☒	The exact sample size (*n*) for each experimental group/condition, given as a discrete number and unit of measurement
☐	☒	A statement on whether measurements were taken from distinct samples or whether the same sample was measured repeatedly
☐	☒	The statistical test(s) used AND whether they are one- or two-sided<br>*Only common tests should be described solely by name; describe more complex techniques in the Methods section.*
☐	☒	A description of all covariates tested
☐	☒	A description of any assumptions or corrections, such as tests of normality and adjustment for multiple comparisons
☐	☒	A full description of the statistical parameters including central tendency (e.g. means) or other basic estimates (e.g. regression coefficient) AND variation (e.g. standard deviation) or associated estimates of uncertainty (e.g. confidence intervals)
☐	☒	For null hypothesis testing, the test statistic (e.g. *F*, *t*, *r*) with confidence intervals, effect sizes, degrees of freedom and *P* value noted<br>*Give P values as exact values whenever suitable.*
☒	☐	For Bayesian analysis, information on the choice of priors and Markov chain Monte Carlo settings
☐	☒	For hierarchical and complex designs, identification of the appropriate level for tests and full reporting of outcomes
☐	☒	Estimates of effect sizes (e.g. Cohen's *d*, Pearson's *r*), indicating how they were calculated

Our web collection on statistics for biologists contains articles on many of the points above.

Software and code

Policy information about availability of computer code

Data collection	BDRhapsody Single-CellAnalysisSystem, NovaSeq 6000
Data analysis	DoubletFinderR software package (version 1.2.2), Seurat R software package (version 4.0.0), R packageSeurat (version4.0.0),SingleR (version 1.2.4), R package GSVA (version1.38.2), R package msigdbr (version 7.4.1), clusterProfiler (V.4.4.4), R package Monocle2 (version 2.16.0), R package CellChat (version1.1.3), CellphoneDB (version2.0.0), GraphPad Prism10.0, R software (version4.0.5), Aperio ImageScope (version 12.3.3.7014), SpectroFlo software (version 3.2.1), Trimmomatic (version 0.36), Bowtie2 (version 2.2.9), MEGAHIT (version 1.1.2), prodigal (version 2.6.3), CDHIT (version 4.6.7)

For manuscripts utilizing custom algorithms or software that are central to the research but not yet described in published literature, software must be made available to editors and reviewers. We strongly encourage code deposition in a community repository (e.g. GitHub). See the Nature Portfolio guidelines for submitting code & software for further information.

Data

Policy information about availability of data

All manuscripts must include a data availability statement. This statement should provide the following information, where applicable:

- Accession codes, unique identifiers, or web links for publicly available datasets
- A description of any restrictions on data availability
- For clinical datasets or third party data, please ensure that the statement adheres to our policy

The raw data of scRNA-seq generated in this study have been deposited in the Genome Sequence Archive in National Genomics Data Center database under

accession code HRA006738, which can be publicly accessible at https://bigd.big.ac.cn/gsa-human. The raw data of mNGS generated in this study have been deposited in the Genome Sequence Archive in National Genomics Data Center database under accession code CRA020419, which can be publicly accessible at https://bigd.big.ac.cn/gsa. Source data are provided with this paper.

Research involving human participants, their data, or biological material

Policy information about studies with human participants or human data. See also policy information about sex, gender (identity/presentation), and sexual orientation and race, ethnicity and racism.

Reporting on sex and gender	Our study counted the sex of donors to eliminate the interference of sex on the results. We have displayed the sex information of all donors in the supplementary table and indicated that 'Male and female sex was referred to the biological sex of a baby at birth'
Reporting on race, ethnicity, or other socially relevant groupings	Not involved
Population characteristics	All samples were obtained from Department of Otolaryngology, Capital Institute of Pediatrics, Beijing, China. Pathological sections borrowed from the Department of Pathology, Capital Institute of Pediatrics, Beijing, China. This study was approved by the Ethics Committee Board of Capital Institute of Pediatrics with number SHERLL2021077, and written informed consent was obtained from all participants included in this study. All participants provided samples free of charge.
Recruitment	Recruit based on the age and disease level of the donors.
Ethics oversight	This study was approved by the Ethics Committee Board of Capital Institute of Pediatrics with number SHERLL2021077, and written informed consent was obtained from all participants included in this study. All participants provided samples free of charge.

Note that full information on the approval of the study protocol must also be provided in the manuscript.

Field-specific reporting

Please select the one below that is the best fit for your research. If you are not sure, read the appropriate sections before making your selection.

☒ Life sciences ☐ Behavioural & social sciences ☐ Ecological, evolutionary & environmental sciences

For a reference copy of the document with all sections, see nature.com/documents/nr-reporting-summary-flat.pdf

Life sciences study design

All studies must disclose on these points even when the disclosure is negative.

Sample size	All experiments contain at least 6 samples or 3 replicates
Data exclusions	No data was excluded in this study.
Replication	All attempts at replication were successful.
Randomization	For human research, donors were grouped based on their disease classification and disease level. For the rat experiment, the rats were randomly divided into two groups.
Blinding	Blinding was not applicable to this study.

Reporting for specific materials, systems and methods

We require information from authors about some types of materials, experimental systems and methods used in many studies. Here, indicate whether each material, system or method listed is relevant to your study. If you are not sure if a list item applies to your research, read the appropriate section before selecting a response.

Materials & experimental systems

n/a	Involved in the study
☐	☒ Antibodies
☒	☐ Eukaryotic cell lines
☒	☐ Palaeontology and archaeology
☐	☒ Animals and other organisms
☐	☒ Clinical data
☒	☐ Dual use research of concern
☒	☐ Plants

Methods

n/a	Involved in the study
☒	☐ ChIP-seq
☐	☒ Flow cytometry
☒	☐ MRI-based neuroimaging

Antibodies

Antibodies used

anti-MASA1 (CST, cat#48750t, clone: E7B7T, 1:1000), anti-CD3 (Affiity, cat#DF6594, clone: Polyclonal, 1:4000), anti-IGHG1 (Abcam, cat#ab109489, clone: EPR4421, 1:5000), anti-LYZ (Abcam, cat#ab108508, clone: EPR2994(2), 1:1000), anti-PLD4 (Affiity, cat#DF4294, clone: Polyclonal, 1:2000), anti-KRT18 (Abcam, cat#ab133263, clone: EPR1626, 1:100), anti-S100A9 (CST, cat#72590t, clone: D5O6O, 1:3000), anti-TIGIT (CST, cat#99567t, clone: E5Y1W, 1:500), anti-PTPRC (CST, cat#13917t, clone: D9M8, 1:20000), anti-CD22 (Abcam, cat#ab207727, clone: EPR20061, 1:10000), anti-XCL1 (Abcam, cat#ab302522, clone: EPR26181-30, 1:100), anti-CCL4 (Abcam, cat#ab45690, clone: EP521Y, 1:500), anti-IGHD (Proteintech, cat#CL488-67538, clone: 1D1B12, 1:300), and anti-CNR2 (SANTA, cat#sc-293188, clone: 3C7, 1:5000), CD45AlexaFluor700 (Biolegend, cat#368514, clone: 2D1, 1:20), CD3BV786 (BD, cat#563800, clone: SK7, 1:20), CD8 FITC (Biolegend, cat#344704, clone: SK1, 1:20), intracellular antibody Granzyme K APC (Biolegend, cat#370510, clone: GM26E7, 1:20), Brilliant Violet 605 anti-human CD197 (CCR7) (Biolegend, cat#353223, clone: G043H7, 1:20), Alexa Fluor 647 anti-human CD11a (Biolegend, cat#301218, clone: HI111, 1:20), PE anti-p53 (Biolegend, cat#645805, clone: DO-7, 1:20), Brilliant Violet 421 anti-human CD284 (TLR4) (Biolegend, cat#312811, clone: HTA125, 1:20), R718 Mouse Anti-Human CD25 (BD, cat#752147, clone: 2A3, 1:20), RB780 Mouse Anti-Human FoxP3 (BD, cat#568682, clone: 259D/C7, 1:20), PE Mouse Anti-FoxP1 (BD, cat#564216, clone: JC12, 1:20), BV421 Mouse Anti-Human RANTES (BD, cat#564754, clone: 2D5, 1:20) and Brilliant Violet 605 anti-human TNF (Biolegend, cat#502935, clone: MAb11, 1:20), CD45 APC-CY7 (Biolegend, cat#368518, clone: 2D1, 1:20), CD3 APC (Biolegend, cat#300312, clone: HIT3a, 1:20) and CD20 FITC (Biolegend, cat#302304, clone: 2H7, 1:20).

Validation

Validations of primary antibodies were determined by manufacturers. The antibodies have been verified to be used on humanandmouse samples, and have been quality tested to be used on flow cytometry and immunohistochemistry. The relevant informationwas available on the manufacturer's websites: https://www.abcam.cn/, https://www.biolegend.com/, https://www.cellsignal.cn/, https://www.bdbiosciences.com/zh-cn/, https://www.affbiotech.cn/, https://www.ptgcn.com/.

Animals and other research organisms

Policy information about studies involving animals; ARRIVE guidelines recommended for reporting animal research, and Sex and Gender in Research

Laboratory animals	Sprague–Dawley rats (aged 4–5 weeks)
Wild animals	This study does not include wild animals.
Reporting on sex	This animal experiment design did not consider sex influence, and rats were randomly assigned by sex.
Field-collected samples	The study did not involve samples collected from the filed.
Ethics oversight	The animal experiments were approved by the Research Ethics Committee of Institute of Chinese Materia Medica, China Academy of Chinese Medical Sciences (2024B162) and complied with ethical standards and international conventions on animal experimentation.

Note that full information on the approval of the study protocol must also be provided in the manuscript.

Clinical data

Policy information about clinical studies

All manuscripts should comply with the ICMJEguidelines for publication of clinical research and a completedCONSORT checklist must be included with all submissions.

Clinical trial registration	This study was approved by the Ethics Committee Board of Capital Institute of Pediatrics with number SHERLL2021077, and written informed consent was obtained from all participants included in this study.
Study protocol	We analyzed the epidemiological characteristics and peripheral blood cell index of 1209 pediatric patients diagnosed as AH (1-15 years old), and found that AH was often accompanied by TH and led to specific changes in immune cell types.
Data collection	The data collection situation was presented in the source data file.

Outcomes

The levels of immune cells in the peripheral blood of patients with different AH grades and with/without TH showed various changes, indicating that AH and TH can activate a variety of immune cells. It was therefore important to more precisely characterize the immune microenvironment of AH and TH tissues.

Plants

Seed stocks

No mentioned.

Novel plant genotypes

No mentioned.

Authentication

No mentioned.

Flow Cytometry

Plots

Confirm that:

- [x] The axis labels state the marker and fluorochrome used (e.g. CD4-FITC).
- [x] The axis scales are clearly visible. Include numbers along axes only for bottom left plot of group (a 'group' is an analysis of identical markers).
- [x] All plots are contour plots with outliers or pseudocolor plots.
- [x] A numerical value for number of cells or percentage (with statistics) is provided.

Methodology

Sample preparation

The single-cell suspension of AH and TH was obtained through grinding. The experiments were conducted with 2x106 cells per group. After FC receptor blocking, cell viability staining, and membrane permeabilization, the cells were stained with CD45AlexaFluor700 (Biolegend, 368514, 2D1), CD3BV786 (BD, 563800, SK7), CD8 FITC (Biolegend, 344704, SK1), intracellular antibody Granzyme K APC (Biolegend, 370510, GM26E7), Brilliant Violet 605 anti-human CD197 (CCR7) (Biolegend, 353223, G043H7), Alexa Fluor 647 anti-human CD11a (Biolegend, 301218, HI111), PE anti-p53 (Biolegend, 645805, DO-7), Brilliant Violet 421 anti-human CD284 (TLR4) (Biolegend, 312811, HTA125), R718 Mouse Anti-Human CD25 (BD, 752147, 2A3), RB780 Mouse Anti-Human FoxP3 (BD, 568682, 259D/C7), PE Mouse Anti-FoxP1 (BD, 564216, JC12), BV421 Mouse Anti-Human RANTES (BD, 564754, 2D5) and Brilliant Violet 605 anti-human TNF-α (BD, 502935, MAb11). The samples were analyzed using the Aurora spectral flow cytometer (Cytek, USA) and data analysis was performed using the SpectroFlo software (Cytek, V3.2.1), with single-stained cells used as reference controls for spectral compensation. Moreover, the single-cell suspension of AH and TH was obtained through grinding. the cells were stained with CD45 APC-CY7 (Biolegend, 368518, 2D1), CD3 APC (Biolegend, 300312, HIT3a) and CD20 FITC (Biolegend, 302304, 2H7). B cells and T cells were sorted using BD FACSAria Fusion Flow Cytometer (BD FACSAria SORP).

Instrument

Aurora spectral flow cytometer (Cytek, USA), BD FACSAria Fusion Flow Cytometer (BD FACSAria SORP)

Software

SpectroFlo software (Cytek, V3.2.1)

Cell population abundance

1000000 cells were selected to analysis, and were distinguish positive and negative.

Gating strategy

According to the living cell population of control group to gate the cells.

- [x] Tick this box to confirm that a figure exemplifying the gating strategy is provided in the Supplementary Information.
